# Generation of simple polygons from ordered points using an iterative insertion algorithm

**DOI:** 10.1371/journal.pone.0230342

**Published:** 2020-03-13

**Authors:** Hongyun Zhang, Quanhua Zhao, Yu Li

**Affiliations:** Institute for Remote Sensing Science and Application, School of Geomatics, Liaoning Technical University, Fuxin, Liaoning, China; Universidad Veracruzana, MEXICO

## Abstract

To construct a simple polygon from a set of plane points, we propose an iterative inserting ordered points (IIOP) algorithm. Using a given a set of ordered non-collinear points, a simple polygon can be formed and its shape is dependent on the sorting method used. To form such simple polygons with a given set of plane points, the points must first be ordered in one direction (typically, the *x*-axis is used). The first three points in the set are used to form an initial polygon. Based on the formed polygon, new polygons can be iteratively formed by inserting the first point of from among the remaining set of points, depending on line visibility from that point. This process is carried out until all the points are inserted into the polygon. In this study, we generated 20, 50, and 80 plane points and used the proposed method to construct polygons. Experimental results show that these three polygons are all simple polygons. Through theoretical and experimental verification, we can concluded that when given a set of non-collinear points, a simple polygon can be formed.

## Introduction

Constructing a simple polygon from a set of plane points is an important process in computational geometry [1] as simple polygons are widely used in computer graphics, image processing, entity construction, and other fields [2–4]. However, the fundamental problem of constructing simple polygons from a set of discrete points is still under investigation.

Currently, several methods are being used to construct a simple polygons from a set of discrete points, such as the polar coordinate sorting [5], dichotomy sorting [6], convex-hull construction [7], and visual region [8] methods. In the dichotomy sorting method [5], two outer points are determined from the maximum and minimum values of *x*-axis (or *y*-axis) projection. The line connecting the two points can divide the set of points into two subsets. The points in these two subsets are sorted according to the value of *x*-axis (or *y*-axis) projection and sequentially connected to form a polygon. In the polar coordinate sorting method [6], a point is randomly selected from a given set as the origin of the polar coordinate system. Afterward, the angles of all the vectors formed by the origin and other points are calculated, and all the points are ordered according to the included angles. The sorted points are then sequentially connected to form a polygon. In the convex-hull construction method [7], a convex hull is first constructed from a given set of points. Here, iteration is started with the convex hull as the initial polygon. In each iteration, a point is randomly selected from the remaining set of points and inserted into the original polygon to form a new polygon. Finally, in the triangle-based construction method [8], an initial triangle is constructed from three randomly selected points and is considered as the initial polygon. The iterative process entails the gradual insertion of the remaining points in the polygon. Although the methods listed above have yielded good experimental results in the process of polygon construction some problems remain. For instance, during polygon construction, invalid points may be encountered. Additionally, it is impossible to prove that a simple polygon can be formed using a given set of discrete points. To solve these problems, in this study, we developed an iterative inserting ordered points (IIOP) algorithm and verified its feasibility of this algorithm theoretically and experimentally. We demonstrate that a simple polygon can be formed using a set of discrete points in which all the points are considered as polygon nodes.

The rest of this article is organized as follows. After defining some functions used in the polygon construction algorithm in the next section, we describe the process of polygon construction and theoretically demonstrate its feasibility. Later, we shall discuss several experimental results and finally present our main conclusions.

## Preliminary definitions

### Simple polygon

Consider a set of *n* points. These points can act as the nodes of a polygon ***G***, if ***G*** satisfies the following conditions.

1. A node connects only two lines.
2. Arbitrary non-adjacent lines do not intersect with each other.
3. There is no loop in the closed geometry.

Under such conditions, ***G*** can be defined as a simple polygon [9].

**Fig 1:**
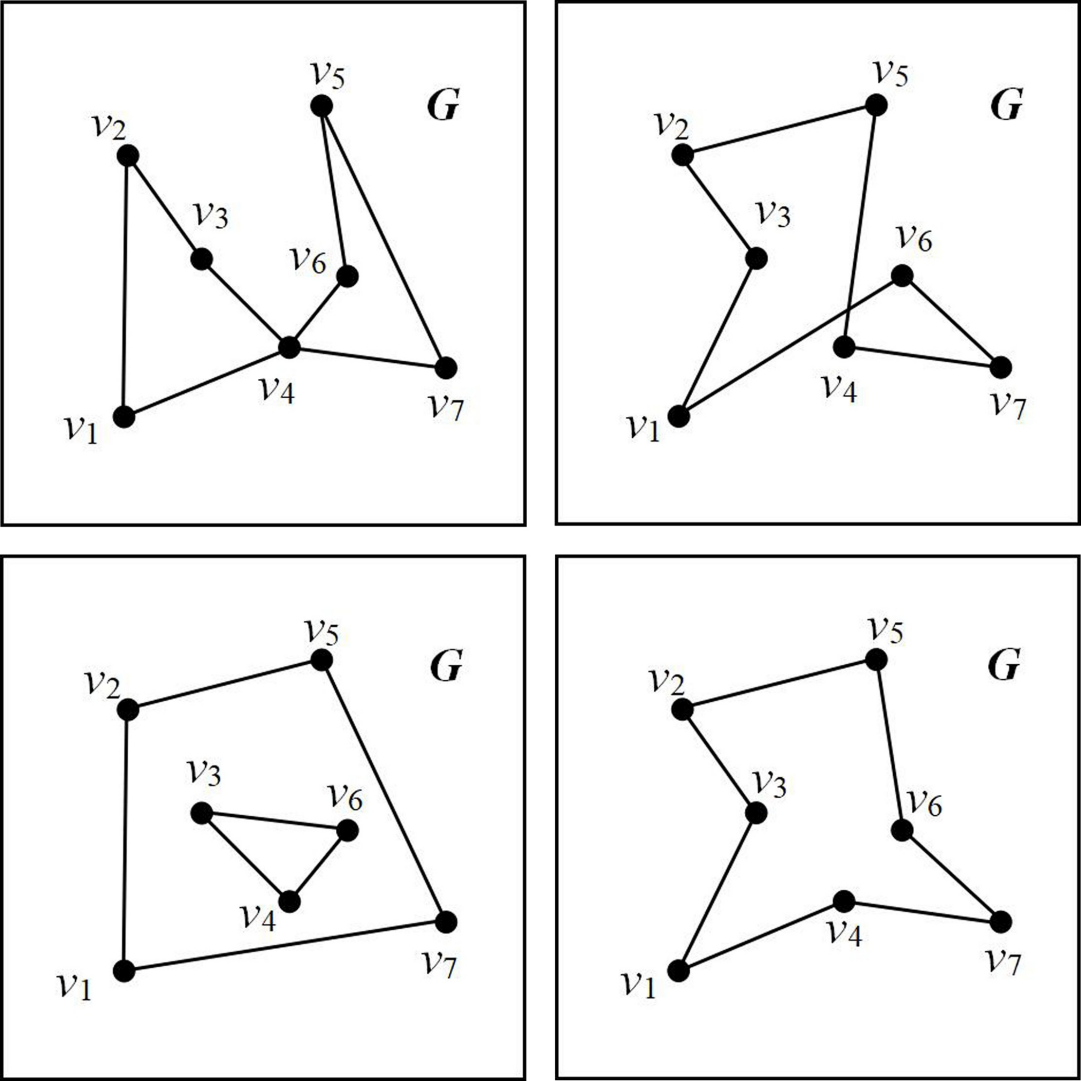
Various types of polygons. (a)-(d) Polygons with different geometries.

Fig 1 show a polygon ***G*** with a set of 7 nodes: this node set can be denoted as ***v*** = {*v*_1_, *v*_2_, *v*_3_, *v*_4_, *v*_5_, *v*_6_, *v*_7_}. In Fig 1(A)–1(D), each polygon has a different geometry and the polygons in Fig 1(A)–1(C) are not simple. In Fig 1(A), point *v*4 connects four lines (*v*_1_*v*_4_, *v*_3_*v*_4_, *v*_4_*v*_6_, and *v*_4_*v*_7_) and hence does not satisfy Condition 1. In Fig 1(B), lines *v*_1_*v*_6_ and *v*_4_*v*_5_ intersect, thus nullifying Condition 2. Meanwhile, Fig 1(C) does not satisfy Condition 3. However, because the polygon in Fig 1(D) satisfies all the conditions listed above, it can be classified as a simple polygon.

### Visibility of a polygon line from a point

Given a polygon and a point outside of the polygon, if a node of the polygon is visible from the point, then a line formed between the node and point does not cross any other polygon line. If and only if a polygon line is visible for the point, the two endpoints of this line are visible for the point [10–12].

**Fig 2:**
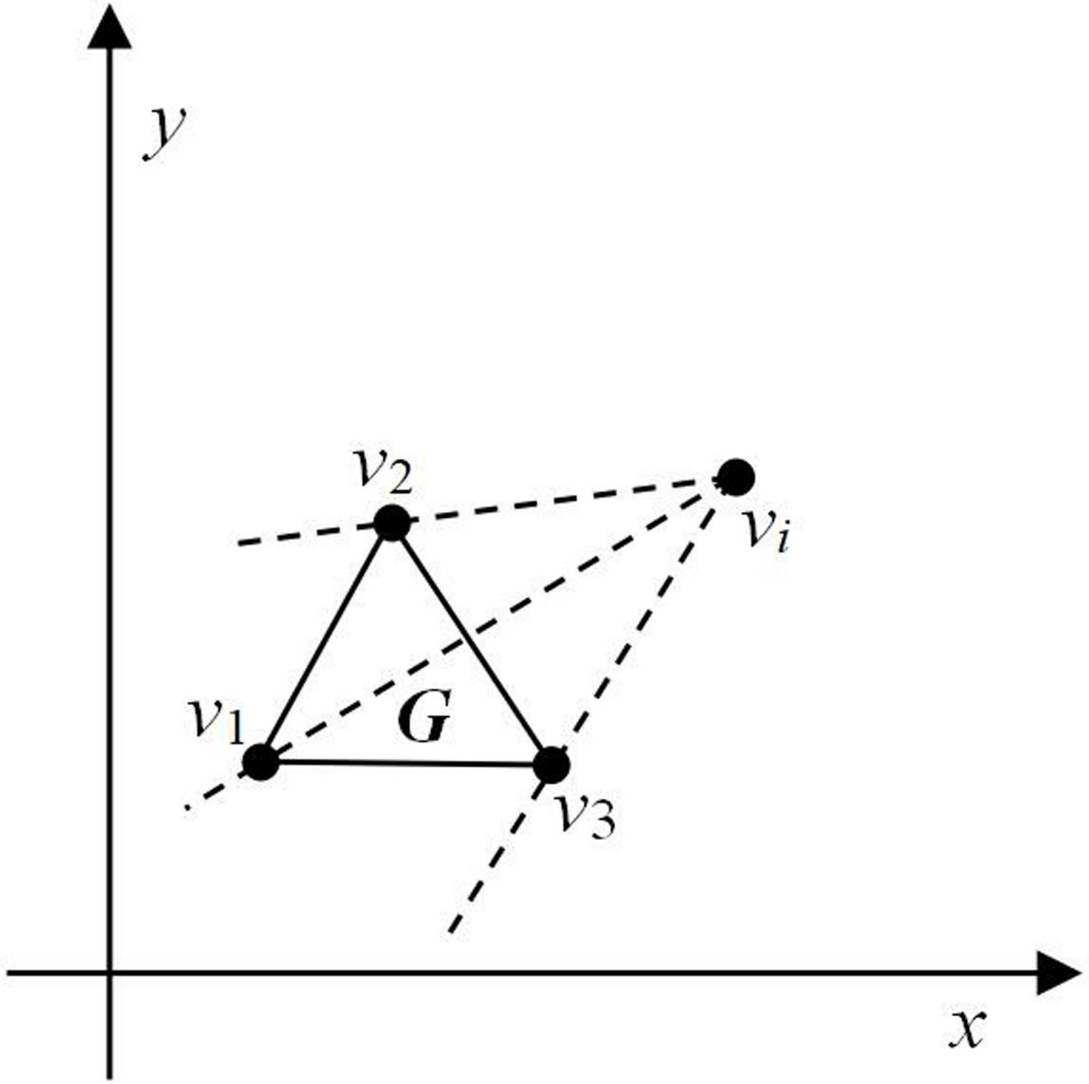
Example of line-to-point visibility.

Fig 2 shows a polygon ***G*** (Δ*v*_1_*v*_2_*v*_3_) and a point *v*_*i*_ outside it. When the point *v*_*i*_ and node *v*_2_ are connected we can see that the new line *v*_*i*_*v*_2_ does not intersect with any other line of the polygon ***G*** and similar is the case with line *v*_*i*_*v*_3_. Thus, we can infer that line *v*_2_*v*_3_ is visible from point *v*_*i*_. When point *v*_*i*_ is connected with node *v*_1_, the new line *v*_*i*_*v*_1_ intersects with line *v*_2_*v*_3_. Therefore, lines *v*_1_*v*_2_ and *v*_1_*v*_3_ are invisible from point *v*_*i*_.

### Line-to-line occlusion at a given point

Assume that there are two lines (*v*_1_*v*_2_ and *v*_3_*v*_4_) and one point (*v*_*i*_). The two lines do not intersect (or only intersect at the endpoints). With respect to point *v*_*i*_, line *v*_1_*v*_2_ occludes line *v*_3_*v*_4_ if and only if the following conditions are satisfied.

1. There is a ray *t* starting at point *v*_*i*_ and it intersects with both lines *v*_1_*v*_2_ and *v*_3_*v*_4_, at different points.
2. If the intersections of ray *t* with lines *v*_1_*v*_2_ and *v*_3_*v*_4_ are *s*_*i*_ and *s*_*j*_ (*i ≠ j*), respectively, then *d*(*v*_*i*_, *s*_*i*_) < *d*(*v*_*i*_, *s*_*j*_) [13–14].

**Fig 3:**
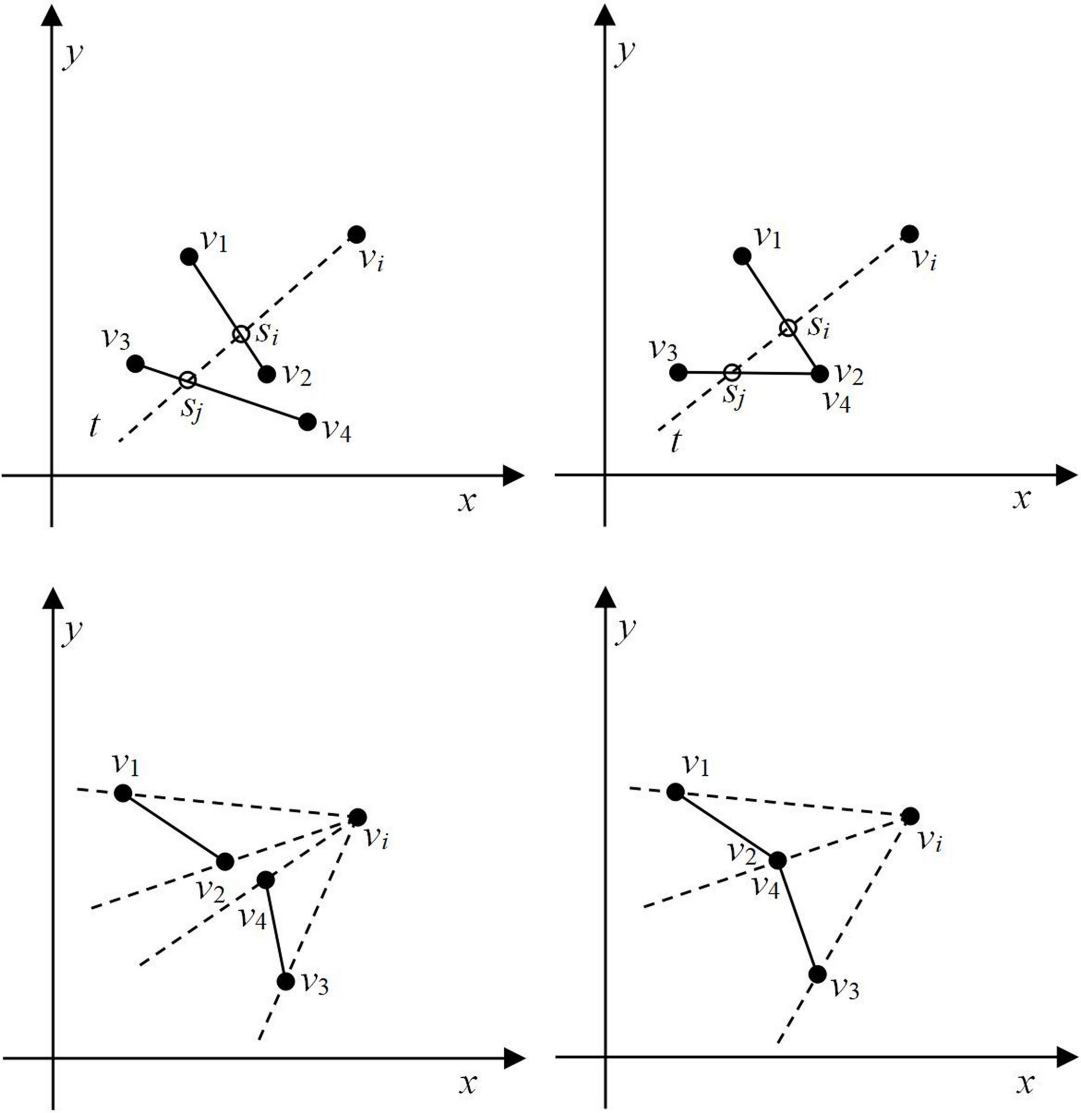
Examples of occlusion. (a) Occlusion with no intersection, (b) occlusion with intersection, (c) no intersection & no occlusion, and (d) no occlusion with intersection.

Fig 3(A) shows two disjoint lines *v*_1_*v*_2_ and *v*_3_*v*_4_. A ray *t* starts at point *v*_*i*_ and intersects with lines *v*_1_*v*_2_ and *v*_3_*v*_4_ at points *s*_*i*_ and *s*_*j*_, respectively, and thus, it satisfies Condition 1. Additionally, we can see that *d*(*v*_*i*_, *s*_*i*_)<*d*(*v*_*i*_, *s*_*j*_), which satisfies Condition 2; hence, it can be inferred that line *v*_1_*v*_2_ occludes *v*_3_*v*_4_ at point *v*_*i*_. Fig 3(B) shows two lines with intersecting endpoints: here, points *v*_2_ and *v*_4_ represent the same point. Moreover, ray *t* intersects lines *v*_1_*v*_2_ and *v*_3_*v*_4_ at points *s*_*i*_ and *s*_*j*_ (*i ≠ j*), respectively, and *d*(*v*_*i*_, *s*_*i*_) < *d*(*v*_*i*_, *s*_*j*_). Therefore, line *v*_1_*v*_2_ occludes *v*_3_*v*_4_ at point *v*_*i*_. Fig 3(C) shows two disjoint lines *v*_1_*v*_2_ and *v*_3_*v*_4_. There is no ray starting at point *v*_*i*_ that can intersect lines *v*_1_*v*_2_ and *v*_3_*v*_4_ simultaneously. Hence, it does not satisfy Condition 1 and there is no occlusion. Fig 3(D) shows two lines with intersecting endpoints. Although there is a ray starting at point *v*_*i*_ that can simultaneously intersect lines *v*_1_*v*_2_ and *v*_3_*v*_4_, intersection occurs at the same point. Therefore, Condition 1 is not satisfied and there is no occlusion.

### Generation of a simple polygon

Consider a set of ordered points ***v*** = {*v*_*i*_ (*x*_*i*_, *y*_*i*_); *i* = 1, …, *n*}, in which no two points are coincident and no three points are collinear. In this point set, *i* is the index of points, *v*_*i*_ is used to indicate the point *i*, *n* is the number of points in the set (usually *n* > 3), and (*x*_*i*_, *y*_*i*_)⊰***R***^2^ represent the coordinates of point *i*. For *v*_*i*_ and *v*_*i*+1_ (*i* = 1, . . . ., *n*—1), if the *x* coordinates are the same *x*_*i*+1_ = *x*_*i*_, then *x*_*i*+1_ = *x*_*i*_; ohterwise *x*_*i*+1_ > *x*_*i*_.

**Fig 4:**
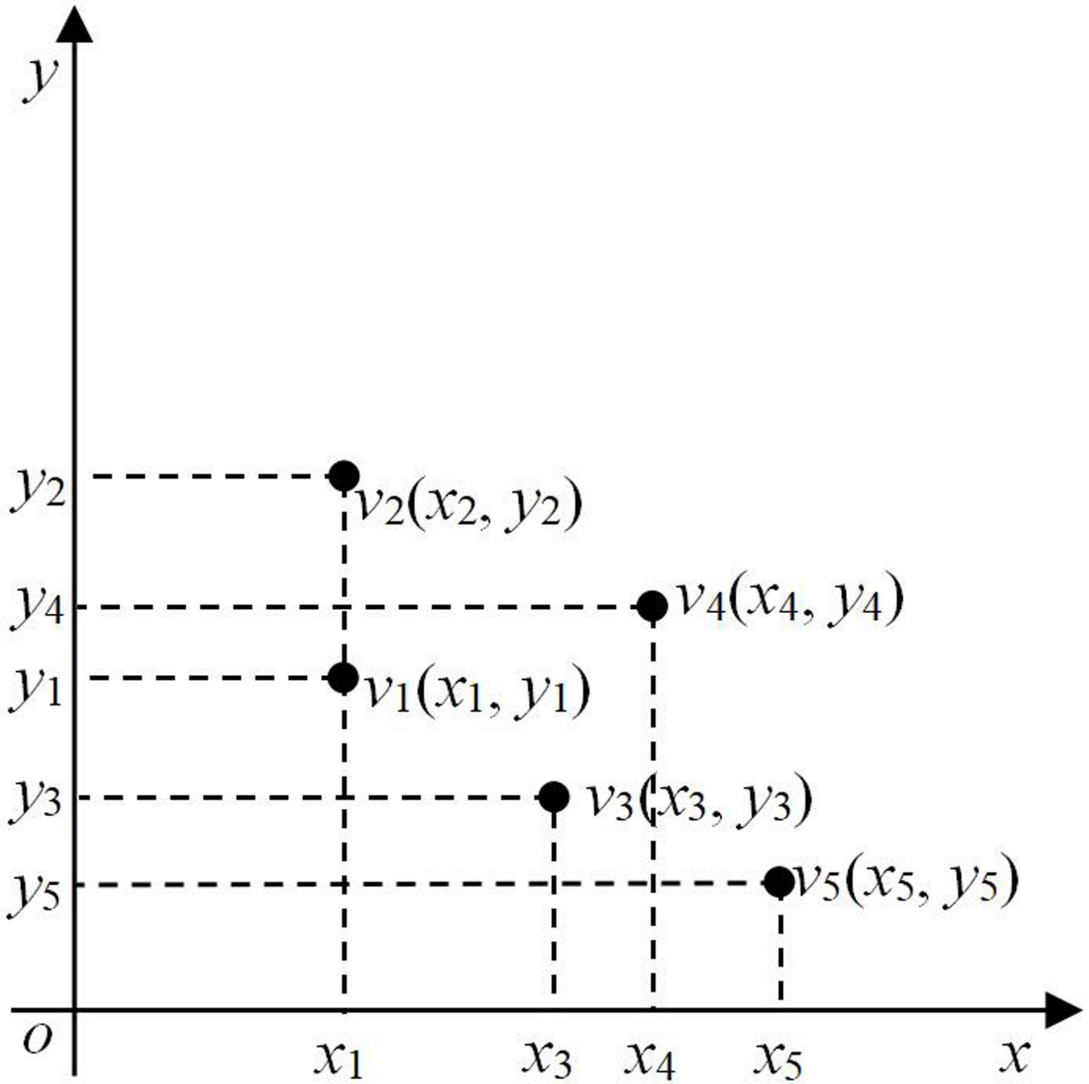
Coordinate axes graph.

Usually, we regard *x*-axis as the horizontal axis, *y*-axis as the vertical axis perpendicular to the *x*-axis, and the intersection of the two axes as origin *o* (Fig 4). There are five ordered points in this figure And their set can be represented as ***v*** = {*v*_1_, *v*_2_, *v*_3_, *v*_4_, *v*_5_}. After sorting, *x*_1_ ≥ *x*_2_ ≥ *x*_3_ ≥ *x*_4_ ≥ *x*_5._ Moreover, when *x*_1_ = *x*_2_, *y*_2_ > *y*_1_. Because the *x*-coordinates of all the points are different, their *y*-coordinates were not considered when ordering.

### Polygon-generation process

(1) Initialization. From a given set of ordered points ***v*** = {*v*_*i*_, *i* = 1, …, *n*}, we select the first *nc*^(0)^ points (usually 3) to construct a polygon ***v***_***c***_^(0)^ (note that ***v***_*c*_ represents both the polygon and the set of points that compose it). The set of points corresponding to this polygon can be denoted as ***v***_***c***_^(0)^ = {*v*_*ck*_^(0)^, *k* = 1, …, *nc*^(0)^}, where *nc*^(0)^ is the point number of the constructed polygon. The residual points in set ***v*** are included in the remaining set ***v***_***s***_^(0)^ = {***v***/***v***_***c***_^(0)^} = {*v*_*sl*_^(0)^, *l* = 1, …, *n–nc*^(0)^}; set ***v***_***s***_ is still a set of ordered points and the sorting method is consistent with that of set ***v***.

**Fig 5:**
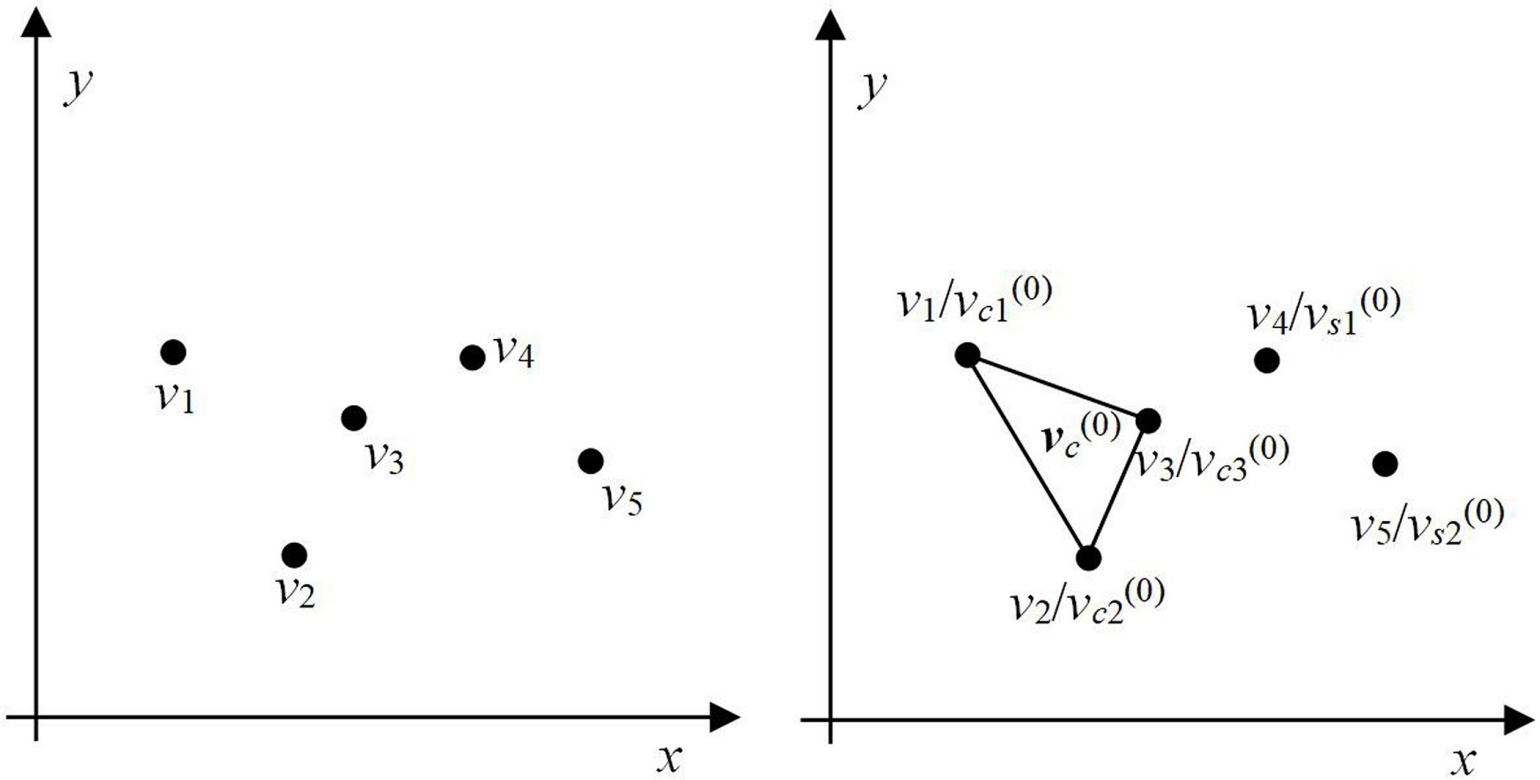
Example of polygon initialization. (a) Random point distribution and (b) polygon initialization.

Fig 5 shows an example of polygon initialization. There are 5 ordered points in the set, i.e., ***v*** = {*v*_1_, *v*_2_, *v*_3_, *v*_4_, *v*_5_} = {*v*_*i*_, *i* = 1, …, *n*}, where *n* = 5. Select the first *nc*^(0)^ points to construct a polygon ***v***_***c***_^(0)^. Set ***v***_***c***_^(0)^ = {*v*_1_/*v*_*c*1_^(0)^, *v*_2_/*v*_*c*2_^(0)^, *v*_3_/*v*_*c*3_^(0)^} = {*v*_*ck*_^(0)^, *k* = 1, …, *nc*^(0)^}, where *nc*^(0)^ = 3 and the symbol / denotes the same point. In the remaining set, ***v***_***s***_^(0)^ = {***v*** / ***v***_***c***_^(0)^} = {*v*_4_/*v*_*s*1_^(0)^, *v*_5_/*v*_*s*2_^(0)^}.

(2) Let *τ* be the iteration indicator. Considering point *v*_*s*1_^(*τ*)^ in the remaining set ***v***_***s***_^(*τ*)^ as an insertion point, we can conclude that the point before point *v*_*s*1_^(*τ*)^ is *v*_*nc*_^(*τ*)^ in the set ***v***. We find two lines which connect point *v*_*nc*_^*τ*)^ from the polygon ***v***_***c***_^(*τ*)^ and judging the visibility of these two lines from point *v*_*s*1_^(*τ*)^. Then insert point into the line which is visible from it. If both lines are visible from point *v*_*s*1_^(*τ*)^, one line is selected randomly. The set of points of the new polygon can be rewritten as ***v***_***c***_^(*τ* + 1)^ = {*v*_*ck*_^(*τ* + 1)^, *k* = 1, 2, …, *n*_*c*_^(*τ* + 1)^}, *nc* ^(*τ* + 1)^ = *nc*^(*τ*)^ + 1, and the iteration indicator is *τ* = *τ* + 1.

**Fig 6:**
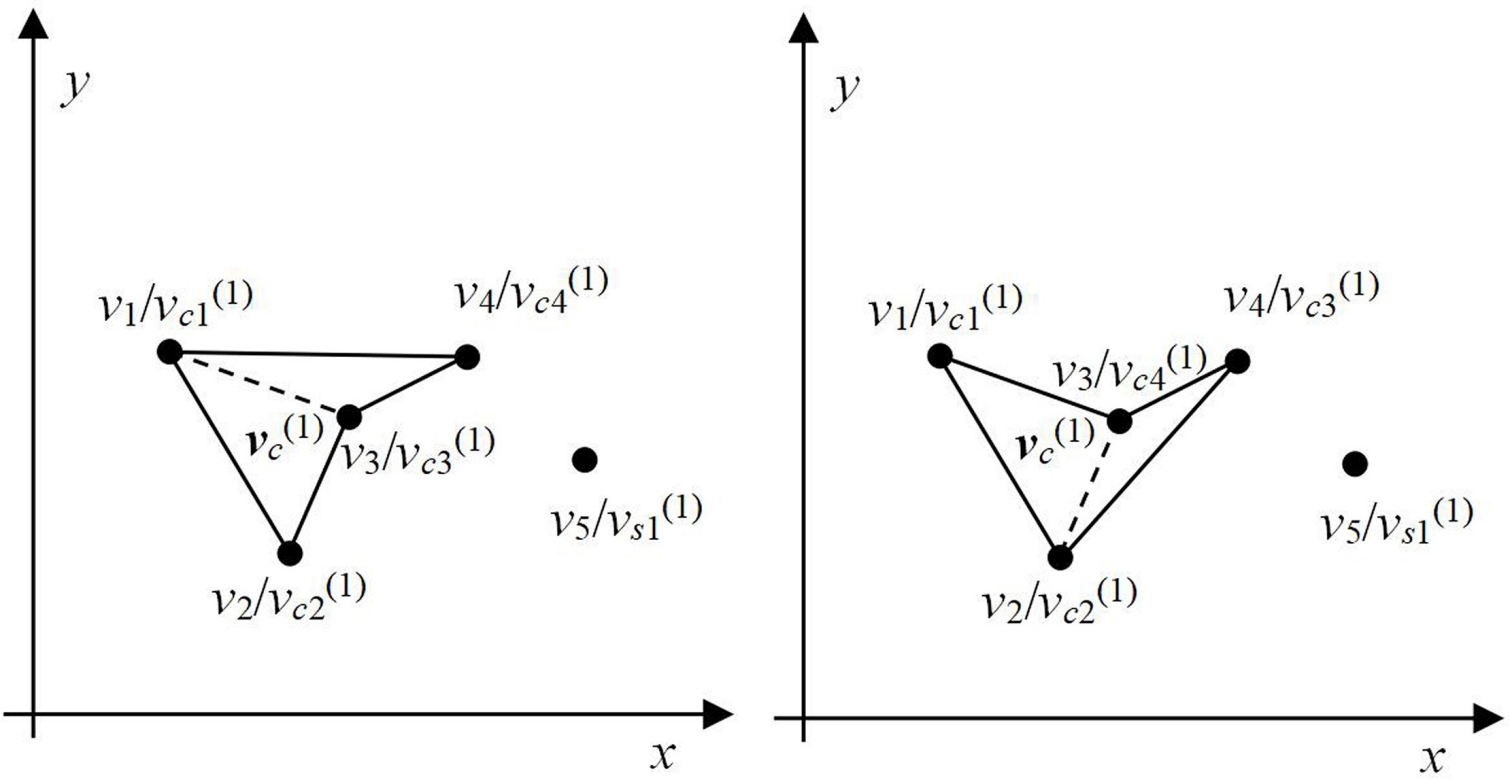
New polygon after one iteration. (a) Polygon 1 and (b) polygon 2.

Polygon ***v***_***c***_^(0)^ is considered as the initial polygon, and the iteration process is started. As shown in Fig 5(B), by judging the visibility of the two lines at *v*_*nc*_^(*τ*)^ = *v*_3_/*v*_*c*3_^(0)^ and *v*_*s*1_^(*τ*)^ = *v*_4_/*v*_*s*1_^(0)^, both *v*_1_*v*_3_ and *v*_2_*v*_3_ are visible from point *v*_*s*1_^(*τ*)^. Therefor, one line is selected randomly and the point *v*_*s*1_^(*τ*)^ is inserted into it; if we select line *v*_1_*v*_3_, the resulting polygon is as shown in Fig 6(A). Consider the set of points ***v***_***c***_^(1)^ = {*v*_1_/*v*_*c*1_^(1)^, *v*_2_/*v*_*c*2_^(1)^, *v*_3_/*v*_*c*3_^(1)^, *v*_4_/*v*_*c*4_^(1)^} = {*v*_*ck*_^(1)^, *k* = 1, …, *nc*^(1)^}, where *nc*^(1)^ = 4 and the remaining set ***v***_***s***_^(1)^ = {***v***/***v***_***c***_^(1)^} = {*v*_5_/*v*_*s*2_^(1)^}. If line *v*_2_*v*_3_ is selected, the resulting polygon is as shown in Fig 6(B). Here, ***v***_***c***_^(1)^ = {*v*_1_/*v*_*c*1_^(1)^, *v*_2_/*v*_*c*2_^(1)^, *v*_4_/*v*_*c*3_^(1)^, *v*_3_/*v*_*c*4_^(1)^} and ***v***_***s***_^(1)^ = {***v***/***v***_***c***_^(1)^} = {*v*_5_/*v*_*s*2_^(1)^}. Evidently, the insertion method affects the order of the set ***v***_***c***_ and polygon geometry. However, it has no impact on the order of the sets ***v*** and ***v***_*s*_.

**Lemma 1** The point before point *v*_*s*1_ is *v*_*nc*_. In the case of two lines *r*_1_ and *r*_2_ on which *v*_*nc*_ is located, at least one line is visible from *v*_*s*1_.

***Proof*:** Assume that the two lines *r*_1_ and *r*_2_ are not visible from *v*_*s*1_. If line *r*_1_ is not visible from *v*_*s*1_, line *r*_2_ occludes *r*_1_. A ray *t* starting from point *v*_*s*1_ intersects lines *r*_1_ and *r*_2_ at points *s*_*m*_ and *s*_*n*_, respectively. According to the definition of occlusion, *d*(*s*_*m*_, *v*_*s*1_) > *d*(*s*_*n*_, *v*_*s*1_). If line *r*_2_ is not visible from *v*_*s*1_, then *r*_1_ occludes *r*_2_. When ray *t* starts at point *v*_*s*1_, it intersects lines *r*_1_ and *r*_2_ at points *s*_*m*_ and *s*_*n*_, respectively. According to the definition of occlusion, *d*(*s*_*n*_, *v*_*s*1_) > *d*(*s*_*m*_, *v*_*s*1_). Contrary to the assumptions, there is no situation in which both lines *r*_1_ and *r*_2_ are not visible from the point *v*_*s*1_. Therefore, it can be demonstrated that at least one line, i.e., either *r*_1_ or *r*_2_ or both, is visible from point *v*_*s*1_.

**Fig 7:**
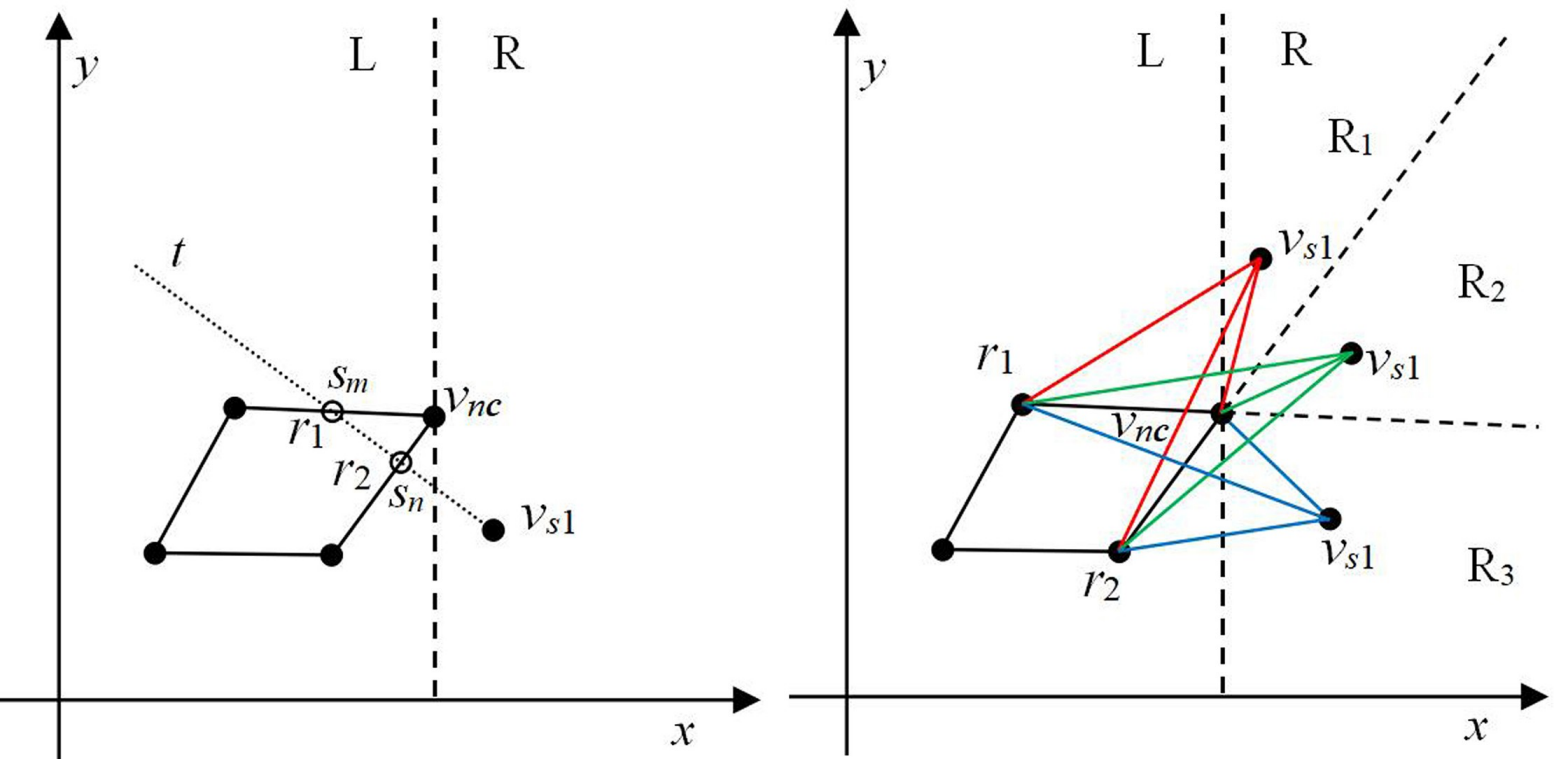
Example of line-to-point visibility. (a) Visibility example and (b) visibility from different regions.

Fig 7 illustrates Lemma 1. As shown in Fig 7(A), the point before point *v*_*s*1_ is *v*_*nc*_. A line passing through *v*_*nc*_ that is perpendicular to the *x-*axis divides the plane into L and R regions. According to the order of the points, point *v*_*s*1_ is in the R region. The ray *t* starting at point *v*_*s*1_ intersects lines *r*_1_ and *r*_2_ at points *s*_*m*_ and *s*_*n*_, respectively. If *d*(*s*_*m*_, *v*_*s*1_) > *d*(*s*_*n*_, *v*_*s*1_), *r*_1_ is not visible from *v*_*s*1_; similarly, if *d*(*s*_*n*_, *v*_*s*1_) > *d*(*s*_*m*_, *v*_*s*1_), *r*_2_ is not visible from *v*_*s*1._ If there is no ray *t* that can simultaneously intersect lines *r*_1_ and *r*_2_ (except line *v*_*s*1_*v*_*nc*_, as shown in Fig 3(D)), both lines are visible from *v*_*s*1_.

Lemma 1 indicates that either *r*_1_ or *r*_2_ or both lines are visible from point *v*_*s*1_. As shown in Fig 7(B), region R can be divided into three parts by extending lines *r*_1_ and *r*_2_, i.e., R = {R_1_, R_2_, R_3_}. When point *v*_*s*1_ is in R_1_, line *r*_1_ is visible and line *r*_2_ is not visible. When point *v*_*s*1_ is in R_3_, line *r*_2_ is visible and line *r*_1_ is not visible from it. When point *v*_*s*1_ is in R_2_, both *r*_1_ and *r*_2_ are visible from it.

(3) The final polygon is generated at an iteration indicator *τ* = *n–* 3. At this time, the number of points in the remaining set is 0. The set of points corresponding to the polygon is ***v***_***c***_^(*n–* 3)^ = {*v*_*ck*_^(*n–* 3)^, *k* = 1, …, *n*} = ***v***.

**Fig 8:**
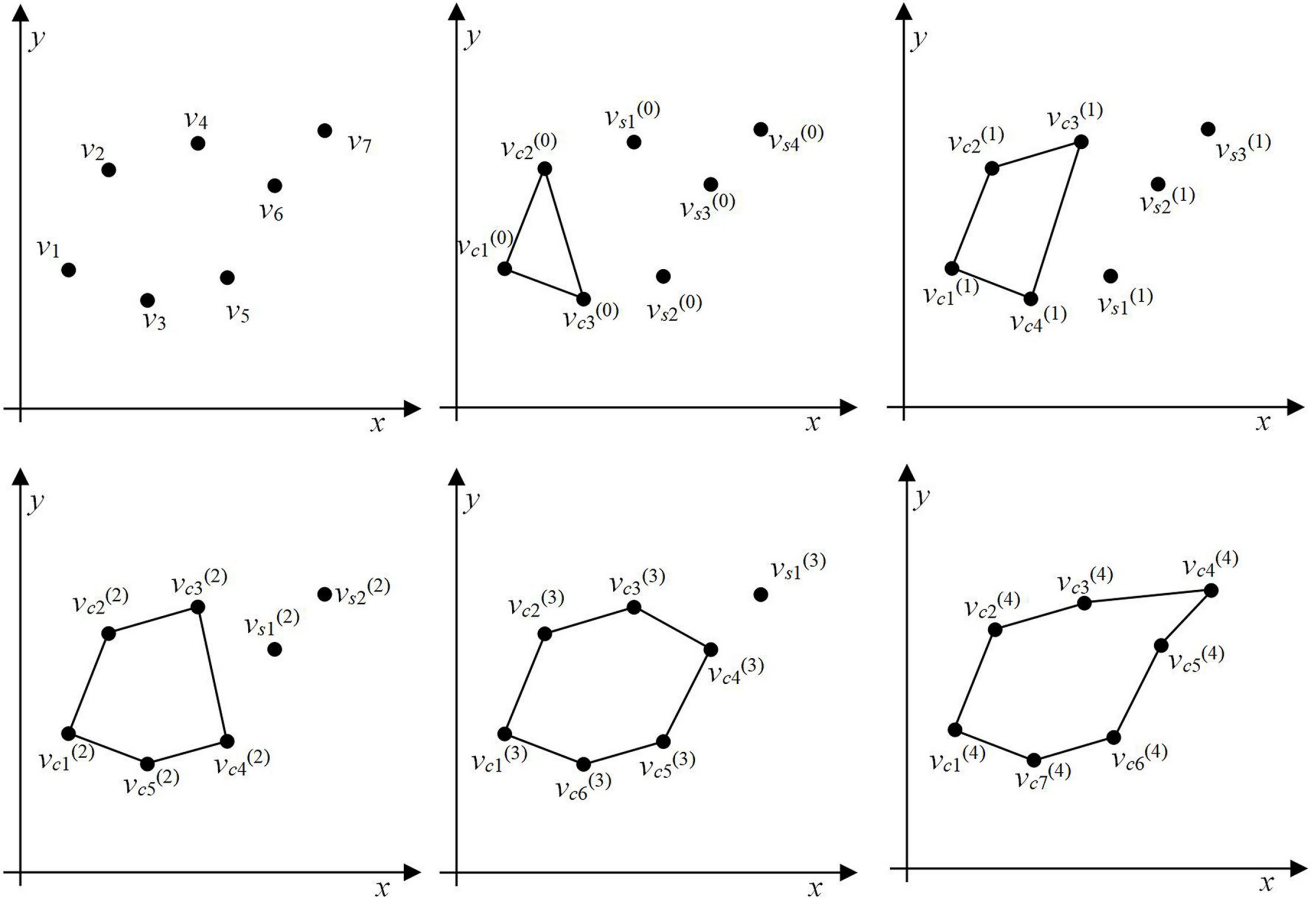
Polygon-generation process. (a) Random point distribution, (b) polygon initialization, (c) result of one iteration, (d) result of two iterations, (e) result of three iterations, and (f) final polygon.

The polygon-generation process is illustrated in Fig 8(A)–8(F). There are seven ordered points that can be denoted as ***v*** = {*v*_1_, *v*_2_, *v*_3_, *v*_4_, *v*_5_, *v*_6_, *v*_7_}. The first three points are connected to form an initial polygon ***v***_***c***_^(0)^ = {*v*_***c***1_^(0)^, *v*_***c***2_^(0)^, *v*_***c***3_^(0)^} and the remaining set of points can be denoted by ***v***_***s***_^(0)^ = {*v*_*s*1_^(0)^, *v*_*s*2_^(0)^, *v*_*s*3_^(0)^, *v*_*s*4_^(0)^}. Consider the first point *v*_*s*1_^(0)^ in ***v***_***s***_^(0)^; the point preceding *v*_*s*1_^(0)^ is point *v*_***c***3_^(0)^. Therefore, we need to judge the visibility of lines *v*_***c***3_^(0)^*v*_***c***2_^(0)^ and *v*_***c***3_^(0)^*v*_***c***1_^(0)^ from *v*_*s*1_^(0)^. The line *v*_***c***3_^(0)^*v*_***c***2_^(0)^ is visible from point *v*_*s*1_^(0)^ and hence, we insert point *v*_*s*1_^(0)^ into the line to form the new polygon ***v***_***c***_^(1)^ = {*v*_***c***1_^(1)^, *v*_***c***2_^(1)^, *v*_***c***3_^(1)^, *v*_***c***4_^(1)^} and the remaining set of points can be written as ***v***_***s***_^(1)^ = {*v*_*s*1_^(1)^, *v*_*s*2_^(1)^, *v*_*s*3_^(1)^}, as shown in Fig 8(C). After the iterative insertion process is completed, the final polygon shown in Fig 8(F) is obtained.

### Polygon-generation algorithm

Given a set of ordered points ***v***, the steps required to generate a polygon can be represented using the following pseudo-codes.

Initialization: Initial polygon ***v***_***c***_ and ***v***_*s*_ is the set of remaining points

For *τ* = 0 to *n–* 3

The number of nodes in polygon ***v***_***c***_^(*τ*)^ is denoted as *nc*^(*τ*)^.
Find two sides of point *v*_*nc*_^(*τ*)^ from polygon ***v***_***c***_^(*τ*)^ and denote them as *r*_1_ and *r*_2_.
Select the first node *v*_*s*1_^(*τ*)^ from ***v***_*s*_^(*τ*)^.
Judge the visibility of *r*_1_ and *r*_2_ from *v*_*s*1_^(*τ*)^.
Insert *v*_*s*1_^(*τ*)^ into *r*_1_ or *r*_2_, whichever line is visible from it.
Generate polygon ***G*** = ***v***_***c***_^(*τ* +1)^.

End

Generate polygon ***G*** = ***v***_*c*_

## Experiments and results

This study proposes a simple polygon-construction method, which proves that in a given set of points, all points can be regarded as polygon nodes and can form a simple polygon. In the current experimental section, the number of simple polygons generated is counted and the criterion of whether all given points can be used as polygon nodes to form simple polygons is used to evaluate the proposed algorithm.

### Relationship between the number of polygons and the number of points

From **Lemma 1** it can be seen that a simple polygon can be formed from a set of points. Additionally, the number of polygons generated is not unique according to the constructing method. From a set of ordered points ***v*** = {*v*_*i*_ (*x*_*i*_, *y*_*i*_); *i* = 1, …, *n*}. Evidently, the first three points *v*_1_, *v*_2_, and *v*_3_ can be used to construct one polygon. Based on our proposed method, point *v*_4_ is inserted into the polygon generated by the first three points, i.e., point *v*_4_ is inserted on either line *v*_1_*v*_3_ or line *v*_2_*v*_3_. Therefore, when both lines are visible from point *v*_4_, two polygons can be formed which implies that a maximum of two polygons can be formed. In the two polygons formed from the four points described above, when point *v*_5_ is inserted, each polygon yields two new polygons. Therefore, a maximum of four polygons can be formed from five points. By analogy, according to the given method, a maximum of 2^*n –* 3^ polygons can be constructed from a set of *n* non-collinear points.

**Fig 9:**
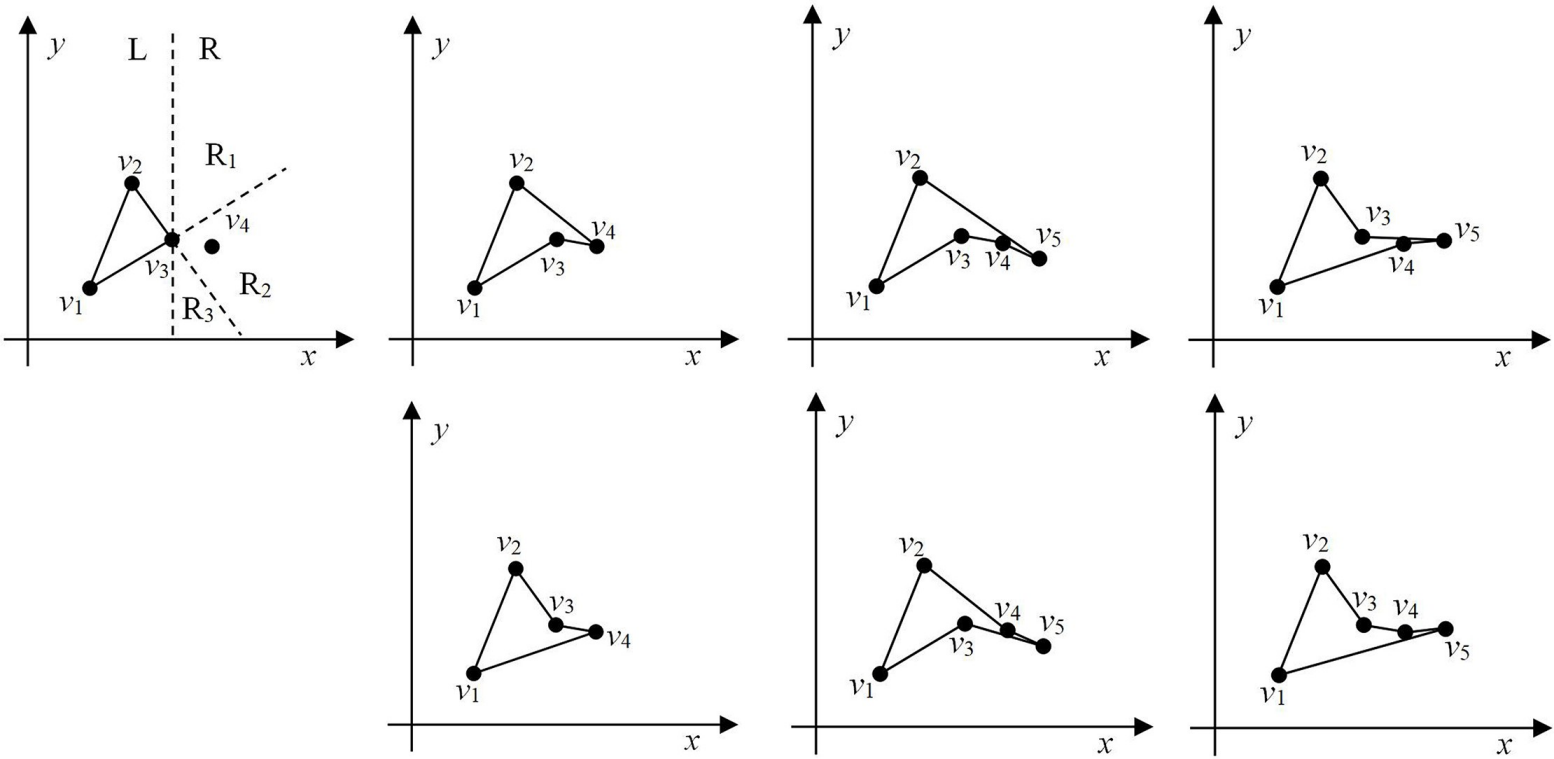
Polygons of different shapes constructed from different number of points. (a) Generation of the initial polygon, (b) polygon constructed from 4 points, (c) polygon constructed from 5 points.

As shown in Fig 9(A), from a set of five ordered points ***v*** = {*v*_1_, *v*_2_, *v*_3_, *v*_4_, *v*_5_}, the first three points *v*_1_, *v*_2_, and *v*_3_ are selected to construct a triangle Δ*v*_1_*v*_2_*v*_3_. subsequently, point *v*_4_ is inserted into the triangle formed by the first three points. Because the points follow a particular order, point *v*_4_ can exist only in the R region, which is divided into three parts denoted as R = {R_1_, R_2_, R_3_}. When point *v*_4_ is present in R_1_ or R_3_, only one of the two lines containing point *v*_3_ is visible from it. Therefore, only one polygon can be constructed. When point *v*_4_ is in R_2_, both lines containing *v*_3_ are visible from it, and hence, it can form two polygons. According to the proposed polygon-construction method, a maximum of two polygons can be constructed using four points (Fig 9(B)). Similarly, when point *v*_5_ is inserted four polygons can be constructed (Fig 9(C)).

### Relationship between polygon shape and point order

In this section, we shall discuss the relationship between the number of points and the number of polygons that can be constructed with the same coordinate order. The influence of different coordinate axis directions on polygon shape when the relative position of a group of points is unchanged is also considered.

**Fig 10:**
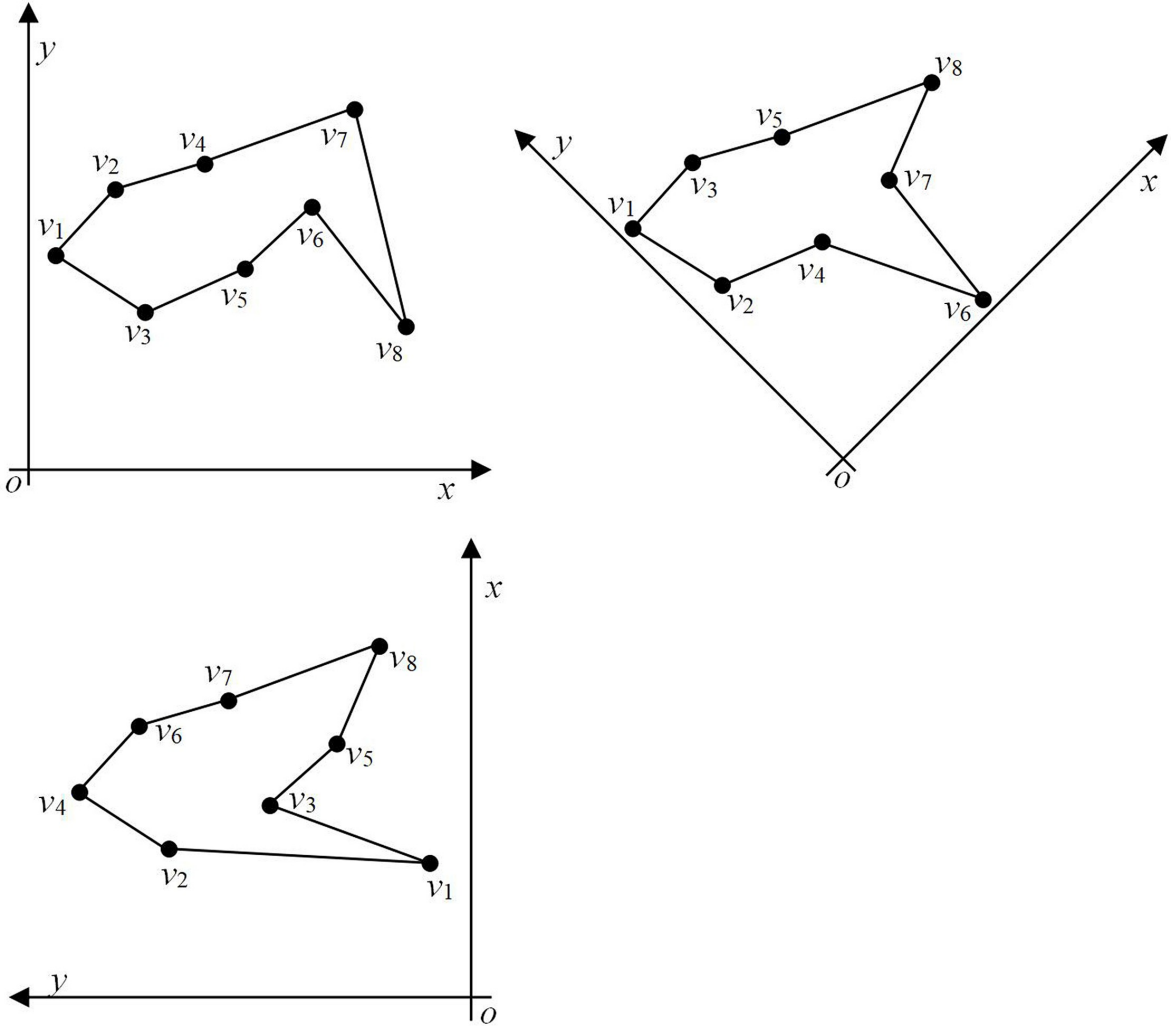
Polygons constructed with coordinate axes in different directions. (a) Horizontal *x*- axis, (b) *x*- axis rotated by 45º to the left, and (c) vertical *x*-axis.

As shown in Fig 10, given a set of points ***v*** = {*v*_*i*_, *i* = 1, …, 8}, the positional relationship between the points remains unchanged. Fig 10(A)–10(C) depict the polygons formed when the *x*-axis direction is horizontal, rotated by 45º to the left, and rotated by 90º to the left, respectively. Note that when the *x*-axis is in different directions, the order between the points as well as the shape of the polygon are different. Therefore, it can be concluded that the shape of the polygon depends on the arrangement of points, which in turn is dependent on the direction of the coordinate axis. However, according to the theoretical proof, given a set of non-collinear points, a simple polygon can be formed regardless of the direction of the coordinates.

### Polygon generation experiment

**Fig 11:**
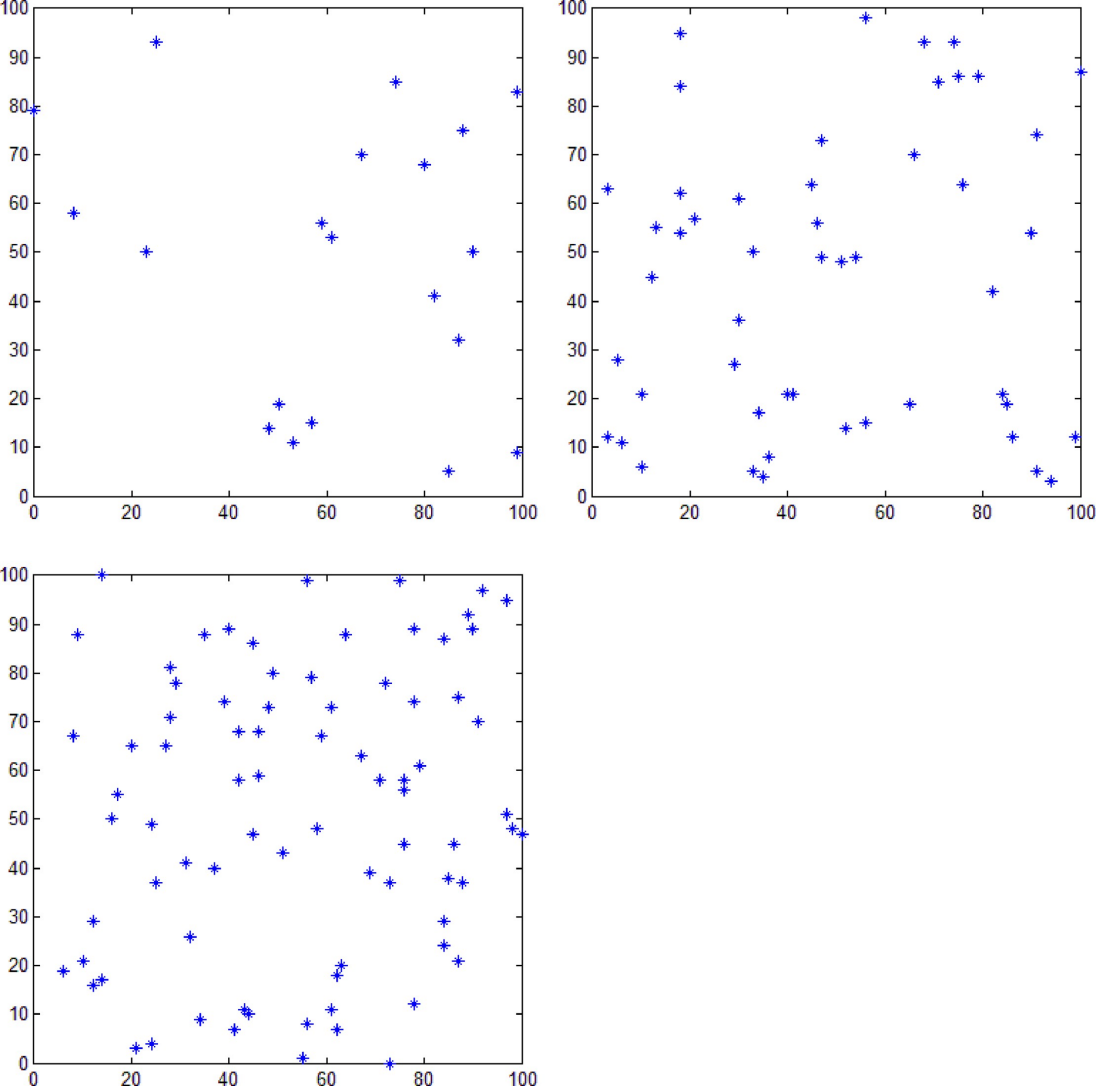
Plane distribution of random points. Plane distribution of (a) 20, (b) 50, and (c) 80 random points.

**Fig 12:**
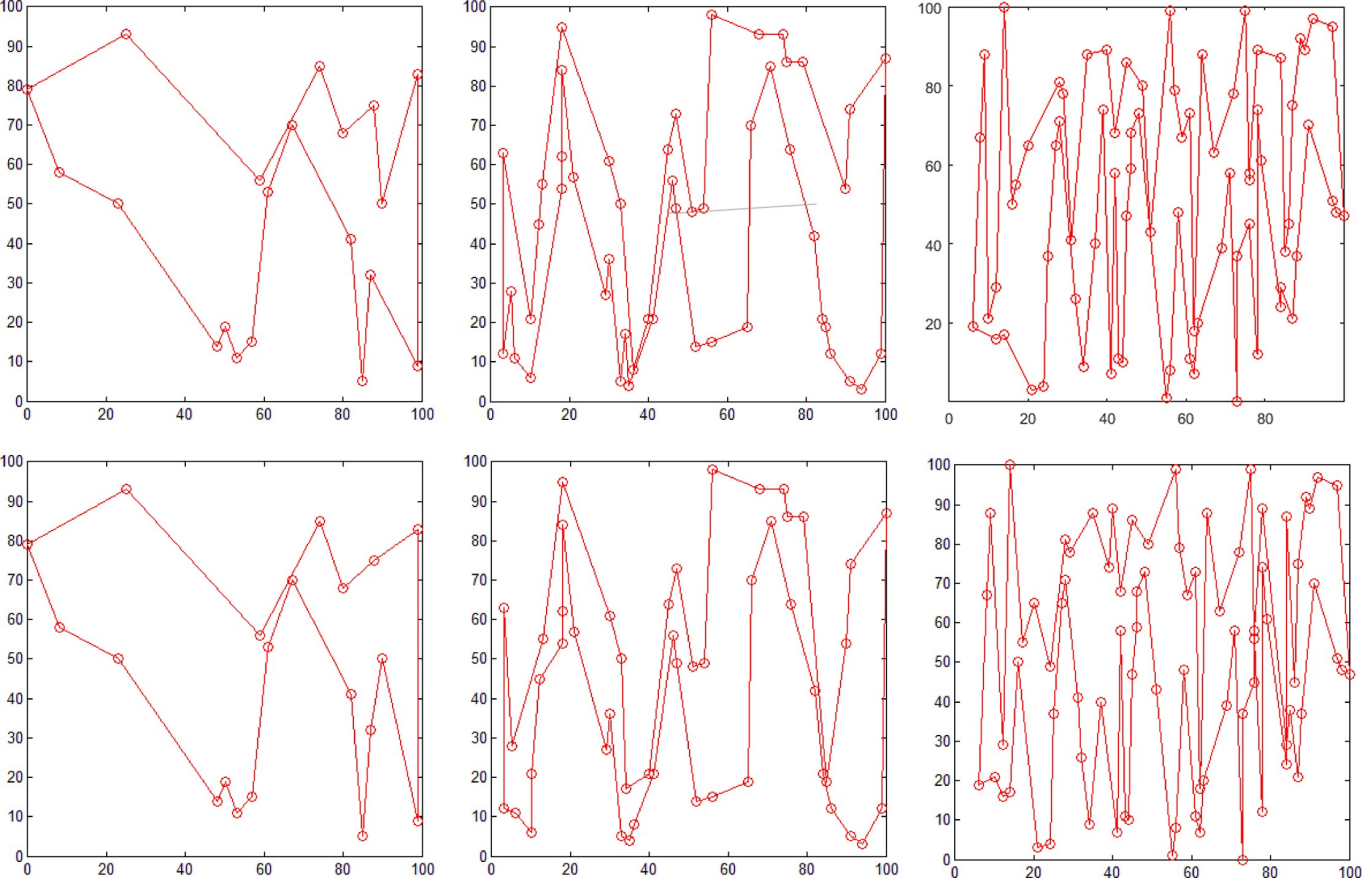
Experimental results of polygon generation. Polygons consisting of (a1)-(a2) 20, (b1)-(b2) 50, and (c1)-(c2) 80 points.

To verify the feasibility of the proposed method, 20, 50, and 80 points were randomly generated and a simple polygon was then generated from each of these sets using the proposed algorithm. Fig 11 shows the plane distribution of 20, 50, and 80 random points. The experimental results are shown in Fig 12. As described in the section on the relationship between number of polygons and number of points, the number of simple polygons generated by the proposed algorithm is not unique. Fig 12 shows two different geometric polygons formed by each group of points. Fig 12(A1)–12(A2), 12(B1)–12(B2) and 12(C1)–12(C2) represent polygons consisting of 20, 50, and 80 points, respectively, and it can be noticed that all the constructed polygons are simple polygons.

**Fig 13:**
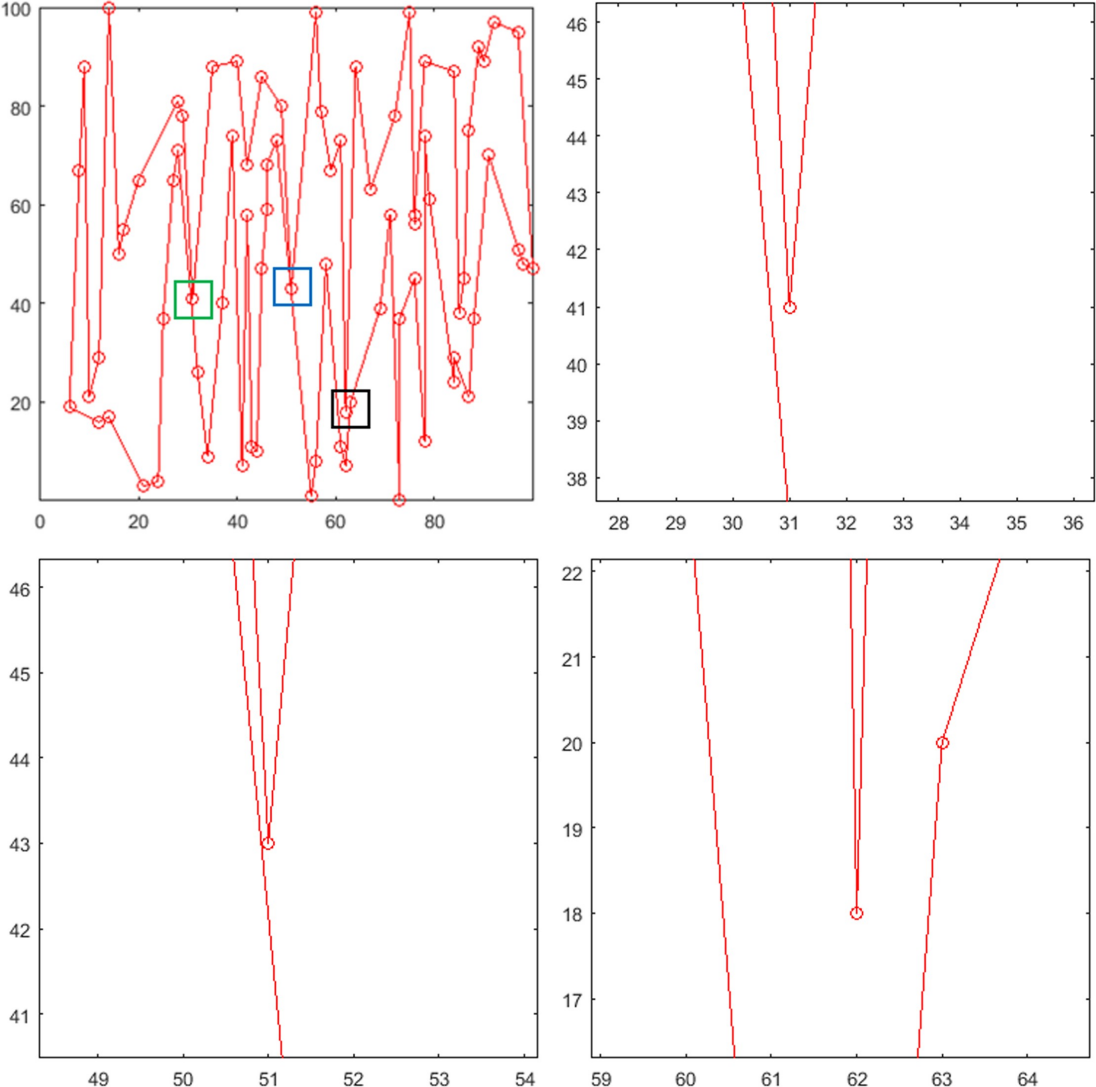
Polygon generation and magnification of partial areas. (a) Polygon consisting of 80 points, (b) enlarged area corresponding to the blue box, (c) enlarged area corresponding to the green box, and (d) enlarged area corresponding to the black area.

Some regions of the polygon with 80 random points cannot be seen clearly. We considered the polygon generated in Fig 12(C1) as an example and enlarged a part of its area (Fig 13). Figs 13(B)–13(D) represent enlarged images of areas in the blue, green, and black boxes in Fig 13 (A), respectively. We can see that the polygon with 80 random points is still a simple polygon. Moreover, the points in the graph are randomly generated, which verifies the feasibility of the proposed method for constructing simple polygons.

### Comparative experiments

**Fig 14:**
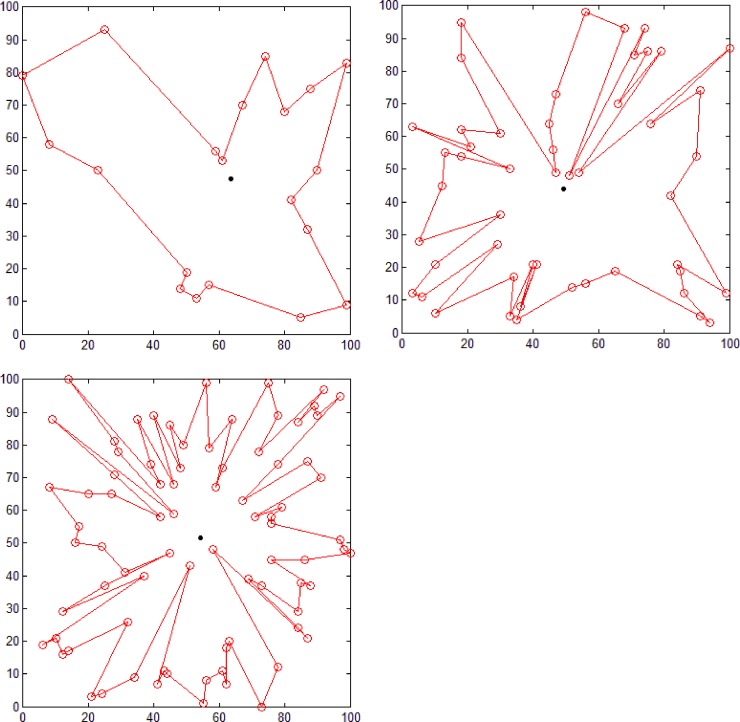
Polygon generation using the polar coordinate sorting method. Polygon consisting of (a) 20 (b) 50, and (c) 80 points.

**Fig 15:**
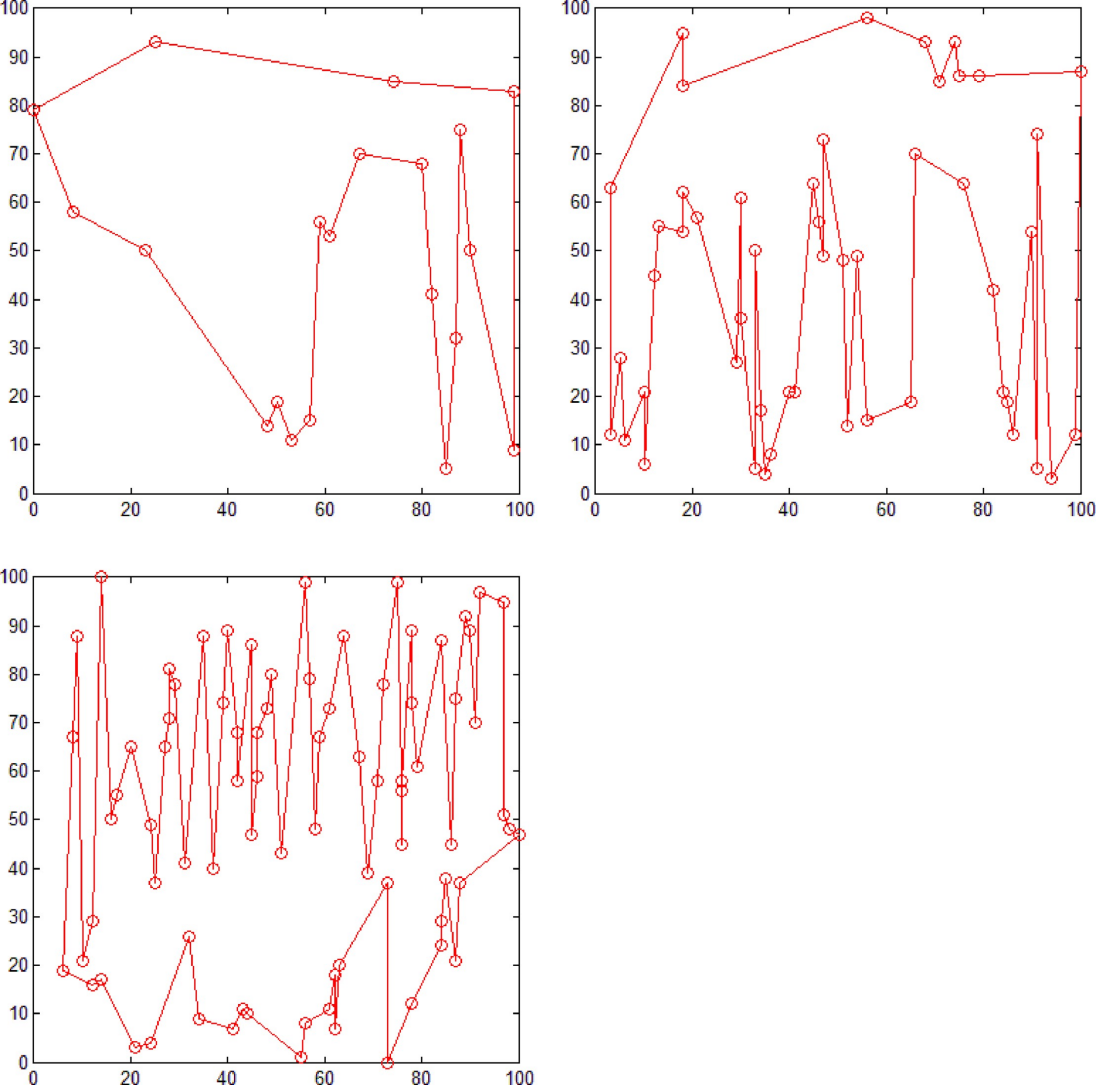
Polygon generating using the dichotomy sorting method. Polygon consisting of (a) 20 (b) 50, and (c) 80 points.

Because both the polar coordinate sorting [5] and dichotomy sorting [6] methods can construct simple polygons using all the given random points as polygon nodes, we compared our proposed method with these two methods for generating simple polygons from three groups of different number of random points (see Fig 11). Fig 14 shows the result obtained using the polar coordinate sorting method, where the black dot represents pole position. According to this algorithm, for a set of random points at a given position, this method can only generate a simple polygon with a fixed geometry. Fig 15 shows the result obtained using the dichotomy sorting method. It can be seen that the number of polygons generated is unique when the direction of a given dichotomy is fixed. However, the proposed method can still construct polygons with different geometric shapes when the coordinate axis direction is given, which implies that the approach proposed in this study is more flexible.

Table 1 shows the time required by the three methods to generate polygons for different random points; it can be inferred that the polar coordinate sorting method is the fastest, while the proposed method is the slowest. However, this slow rate can be attributed to its ability to generate polygons of different geometric shapes for which more time is required.

**Table 1:** Time required to generate a simple polygon.

Time (s)	Number of random points		
	20	50	80
Proposed method	0.25	0.30	0.37
Polar coordinate sorting method	0.07	0.09	0.11
Dichotomy sorting method	0.09	0.12	0.13

The number of simple polygons constructed by the polar coordinate sorting and dichotomy sorting methods is unique, while the number of simple polygons constructed by the convex-hull construction method [7] is non-unique. However, the latter cannot generate a simple polygon using all the given random points as polygon nodes.

**Fig 16:**
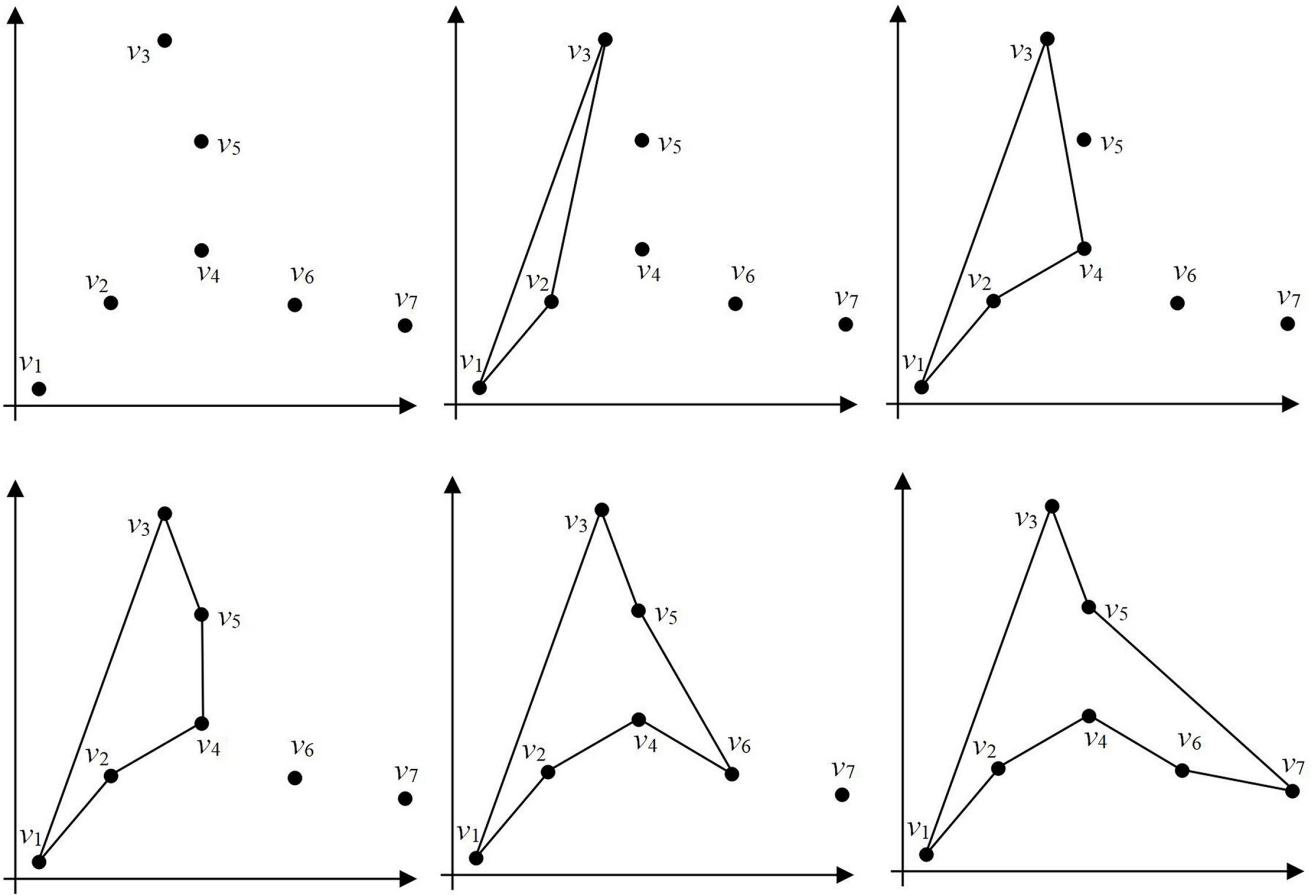
Polygon generation using the proposed method. (a) Random point distribution, (b) polygon initialization, (c) result of one iteration, (d) result of two iterations, (e) result of three iterations, and (f) the final polygon.

Consider a set of seven ordered points ***v*** = {*v*_1_, *v*_2_, *v*_3_, *v*_4_, *v*_5_, *v*_6_, *v*_7_}, as shown in Fig 16(A). The polygon-construction process using the proposed method is shown in Fig 16(B)–16(E) and Fig 16(F) shows the final polygon, which is a simple polygon.

**Fig 17:**
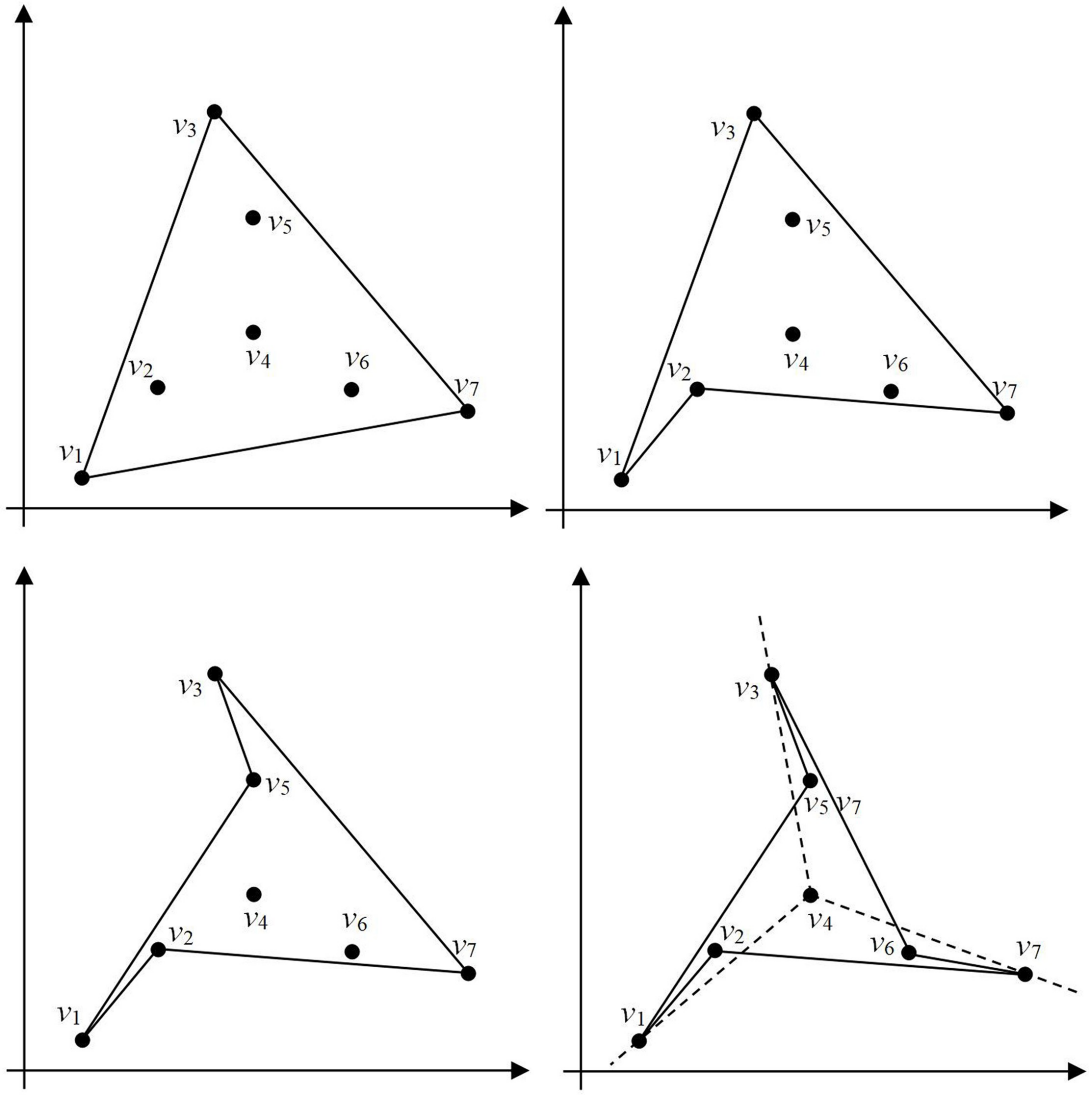
Polygon generation using the convex-hull construction method. (a) Polygon initialization, (b) result of one iteration, (c) result of two iterations, and (d) result of three iterations.

Fig 17 shows the results of polygon generation using the convex-hull construction method. First, the convex hull of a given node set is generated (Fig 17(A)); later, a point is randomly selected from the remaining points and inserted into the line visible from it to form a new polygon. The final polygon is shown in Fig 17(D). However, none of the polygon edges are visible from point *v*_4_. Therefore, in this situation, point *v*_4_ is called an invalid point. Finally, we can infer that polygon construction based on the convex-hull method includes a strong element of randomness owing to which it is impossible to construct a simple polygon with all the points in a given set. Unlike the method proposed in this study, using which a simple polygon can be constructed from a given set of non-collinear points, in the convex-hull construction method, invalid points may occur.

## Conclusion

In this study, we designed an IIOP algorithm to generate a simple polygon from a set of ordered points. Through theoretical and experimental verification of this algorithm, the following three conclusions could be drawn. (1) Given a set of non-collinear points, a simple polygon can be formed. (2) The shape of a simple polygon depends on the sorting method used. (3) The number of polygons is related to the number of random points. When the sorting method is fixed, at least 1 or a maximum of 2^*n –* 3^ simple polygons can be formed, where *n* is the number of non-collinear points in a set.
